# Long-term response to gluten-free diet as evidence for non-celiac wheat sensitivity in one third of patients with diarrhea-dominant and mixed-type irritable bowel syndrome

**DOI:** 10.1007/s00384-016-2663-x

**Published:** 2016-09-30

**Authors:** Christian Barmeyer, Michael Schumann, Tim Meyer, Christina Zielinski, Torsten Zuberbier, Britta Siegmund, Jörg-Dieter Schulzke, Severin Daum, Reiner Ullrich

**Affiliations:** 1 Medical Department, Division of Gastroenterology, Infectiology, and Rheumatology, Campus Benjamin Franklin, Charité—Universitätsmedizin Berlin, Hindenburgdamm 30, 12200 Berlin, Germany; 2 Institute of Clinical Physiology, Campus Benjamin Franklin, Charité—Universitätsmedizin Berlin, Berlin, Germany; 3 Department of Dermatology and Allergy, Allergy-Centre-Charité, Campus Mitte, Charité—Universitätsmedizin Berlin, Berlin, Germany; 4 Department of Medical Microbiology, Immunology, and Hygiene, Technische Universität, Munich, Germany

**Keywords:** Irritable bowel syndrome, Wheat-sensitivity, Gluten-free diet, HLA-DQ2, HLA-DQ8

## Abstract

**Purpose:**

Irritable bowel syndrome (IBS) is common but therapies are unsatisfactory. Food is often suspected as cause by patients, but diagnostic procedures, apart from allergy testing, are limited. Based on the hypothesis of non-celiac wheat sensitivity (WS) in a subgroup of IBS patients, we tested the long-term response to a gluten-free diet (GFD) and investigated HLA-DQ2 or -DQ8 expression as a diagnostic marker for WS in diarrhea-dominant (IBS-D) and mixed-type IBS (IBS-M).

**Methods:**

The response to a GFD served as reference test for WS and HLA-DQ2/8 expression was determined as index test. Patients were classified as responders if they reported complete or considerable relief of IBS symptoms on at least 75 % of weeks over a 4-month period of gluten-free diet. Established questionnaires (IBS-Quality of Life (IBS-QoL), IBS Symptom Severity Scale (IBS-SSS), European Quality of Life-5 Dimensions (EQ-5D)) were used for secondary outcome measures.

**Results:**

Thirty-five patients finished the study. Of these, 12 (34 %) were responders and classified as having WS (95 % CI 21–51 %). HLA-DQ2/8 expression had a specificity of 52 % (95 % CI 33–71 %) and sensitivity of 25 % (95 % CI 8–54 %) for WS. Responders showed improvement in quality of life and symptom scores. At 1-year follow-up, all responders and 55 % of non-responders were still on GFD and reported symptom relief.

**Conclusion:**

Using strict criteria as recommended for IBS studies, about one third of patients with IBS-D or IBS-M are wheat sensitive, with a similar proportion in both IBS types. Expression of HLA-DQ2/8 is not useful as diagnostic marker for WS. Long-term adherence to a GFD is high and can sustain symptomatic improvement.

**Electronic supplementary material:**

The online version of this article (doi:10.1007/s00384-016-2663-x) contains supplementary material, which is available to authorized users.

## Introduction

Irritable bowel syndrome (IBS) is a functional bowel disorder that is characterized by abdominal pain or discomfort in conjunction with altered bowel movements and a lack of biochemical or structural abnormalities when using regular diagnostic procedures [1, 2]. Approximately 4–20 % of the population in western countries are affected during their lifetime [3]. Patients are suffering from low quality of life and reduced social contacts. In particular, primary care physicians are challenged with unsatisfactory treatment options. The social and economic impact is remarkable due to the reduced quality of life and high rates of IBS-related non-productive time and invalidity pension [4–8]. Data from the USA show that IBS accounts for up to 25 % of all patients seen in gastroenterologists’ practice. It is therefore the most common diagnosis made by gastroenterologists and the seventh most prevalent diagnosis made by all physicians [9]. Patients often suspect food but classical food allergy is rarely the reason.

During the noughties, evidence arose that subgroups of IBS patients may be “gluten sensitive” and could therefore respond to a gluten-free diet (GFD) even in the absence of the histological abnormalities characteristic for celiac disease [10–15]. These observations led to the concept of a new disorder that is summarized under the term non-celiac gluten sensitivity or, because wheat components apart from gluten may be involved, more recently non-celiac wheat sensitivity (WS) [16–18].

In this context, we have reported a benefit for patients with diarrhea-dominant IBS who are carrying the HLA-DQ2 allele after being on a GFD for 6 months in an open uncontrolled trial [14]. We therefore speculated that HLA-DQ2 could be a useful marker to identify a subgroup of gluten-sensitive IBS patients who benefit from GFD. Other markers associated with celiac disease like elevated serum anti-gliadin and/or anti-tissue transglutaminase (anti-TTG) IgG or elevated succus anti-gliadin and/or anti-TTG IgA antibodies were also associated with a response to GFD indicating that potential or latent celiac disease could be present in this cohort.

These results required confirmation in a study using established response criteria for IBS therapies. Furthermore, we wondered whether IBS patients without diarrhea could similarly profit from gluten withdrawal as suggested by the significant improvements of IBS symptoms apart from diarrhea [14].

Thus, the aim was to analyze if HLA-DQ2 and-DQ8 are suitable markers for the diagnosis of WS and to evaluate the long-term clinical response to a GFD in an exactly defined cohort of patients with diarrhea-dominant IBS (IBS-D) and mixed-type IBS (IBS-M). For this purpose, we designed a prospective, double-blind phase 3 diagnostic study of HLA-DQ2/8 typing for WS in patients with IBS-D and IBS-M that were put on a GFD for 4 months with a follow-up period of 1 year and analyzed the changes in the scores of IBS- and health-related quality of life (QOL) questionnaires and a visual analog scale (VAS) before and after treatment with GFD.

## Methods

The study was approved by the local ethics committee of the Charité—Universitätsmedizin Berlin under the number EA4/044/11. Written informed consent was obtained from all study participants.

### Study population

Consecutive non-constipated IBS patients with weekly symptoms, above 18 years old, male and non-pregnant female, who were able and willing to follow a 4-month GFD, were included. Upon study entry, other medical conditions presenting with similar symptoms as IBS were excluded on the basis of medical history, physical examination, blood tests (including TSH, ESR and/or CRP, total IgA, anti-tissue-transglutaminase IgA, WBC, hemoglobin, platelets, creatinine, beta-hCG), gastroscopy with duodenal biopsies, abdominal ultrasound, lactose intolerance test, and stool examination on pancreatic elastase and stool culture. Colonoscopy was performed at study entry unless patients already had an earlier colonoscopy because of their symptoms to exclude other diseases like colon cancer or inflammatory diseases, such as ulcerative colitis, Crohn’s disease, or microscopic colitis. Wheat allergy was excluded with a skin test. For inclusion, IBS patients had to fulfill the Rome III criteria for IBS-D or IBS-M based on a diagnostic questionnaire published by the Rome Foundation (IBS module; www.romecriteria.org/pdfs/IBSMode.pdf). According to these criteria, IBS is existent, when abdominal discomfort or pain occurred for at least 3 days per month within the last 3 months (onset of symptoms at least 6 months prior to the diagnosis) and is associated with at least two out of the three following criteria: (i) improvement with defecation, (ii) onset associated with a change in frequency of stool, and/or (iii) onset associated with a change in appearance of stool. Patients not fulfilling these criteria were excluded. Because constipation is no classical feature of celiac disease [19, 20], we did not expect WS in constipated IBS patients and therefore excluded them from the study.

Patients were recruited from the outpatient clinic of the Medical Department, Division of Gastroenterology, Infectiology and Rheumatology (Charité, Campus Benjamin Franklin, Berlin) as well as from nearby gastroenterologists, primary care physicians, and through public advertising in newspapers and public transportation.

### Study protocol

**Fig. 1:**
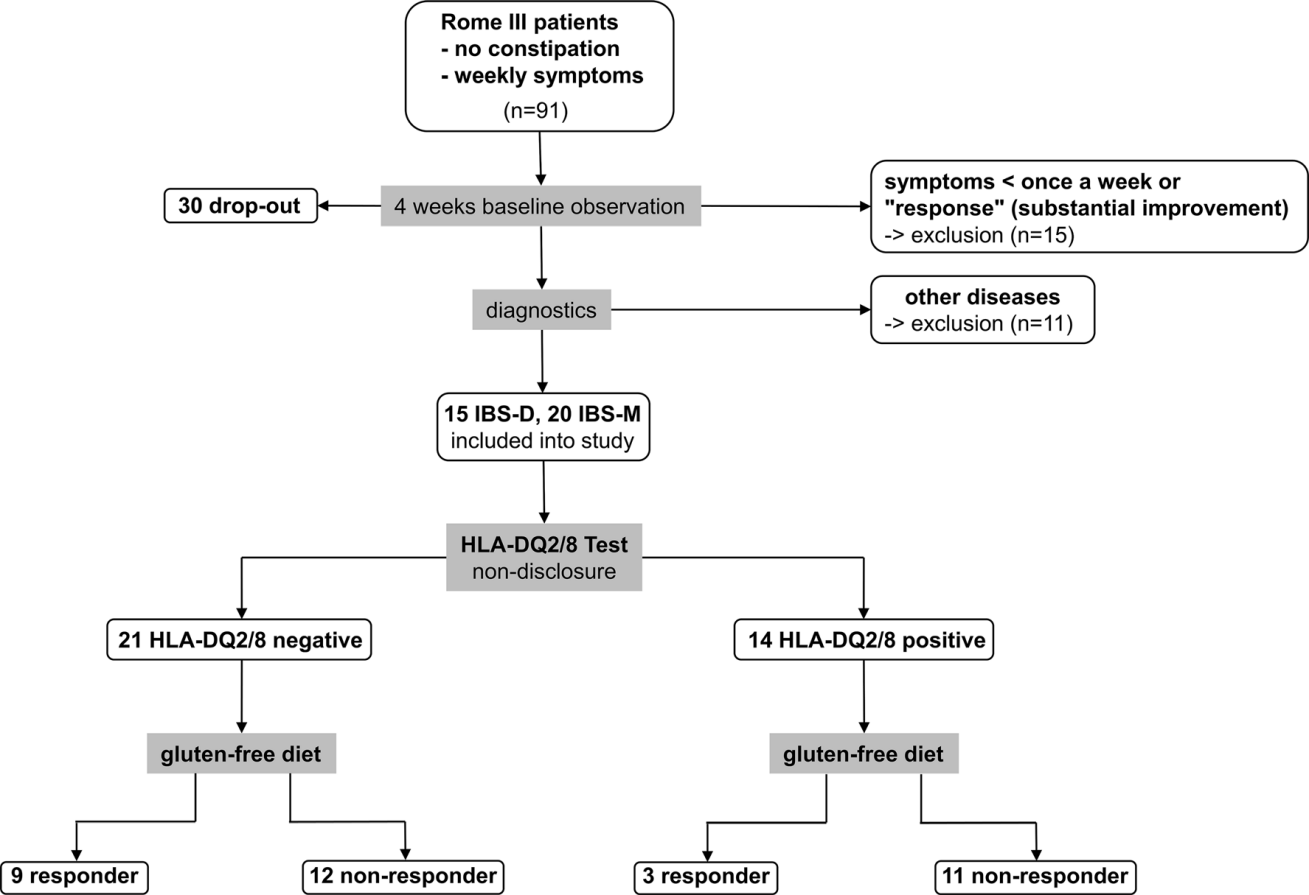
Diagram of the patient flow through the study. Ninety-one Rome III fulfilling patients with self-reported weekly symptoms entered a 4-week observation period during which 45 (49 %) were excluded due to improvement or lack of weekly symptoms (15 patients) and other mainly personal reasons (30 patients). The remaining 46 patients entered a diagnostic stage during which 11 (24 %) were excluded due to a new diagnosis. The remaining 35 patients entered the trial stage. Depicted here is the result of the 75 % criterion (“considerably relieved” or “completely relieved” at 75 % of the time during the GFD). According to this, 12 patients were classified as responders and 23 patients as non-responders with no correlation between response to GFD and HLA-DQ2/8 status

The trial design is depicted in Fig. 1. On the basis of an initial interview, potentially eligible subjects were identified that fulfilled the Rome III criteria and reported weekly symptoms. They entered a 4-week observation period without any intervention. At the end of each week the “Subject’s Global Assessment (SGA) of relief” was determined by phone. The SGA of relief is a well-established measure and has been validated before [21, 22]. It consists of one question (“Please consider how you felt this past week with regard to your IBS, in particular your overall wellbeing, and symptoms of abdominal discomfort, pain and altered bowel habit. Compared to the way you usually felt before entering the study, how would you rate your relief of symptoms during the past week?”) and offers the following five different answers: (1) completely relieved, (2) considerably relieved, (3) somewhat relieved, (4) unchanged, or (5) worse. Patients who answered “unchanged” or “worse” at all four occasions received diagnostics to exclude other medical conditions before they were included into the study.

Patients received two dietary consultations by a professional dietician at an interval of 2 weeks: the first consultation to instruct them how to follow the GFD, the second consultation to ascertain the correct implementation of the diet. Thereafter, patients were monitored for 4 months by weekly assessment of the SGA of relief by phone calls. To evaluate changes in IBS symptoms and quality of life before and after the dietary intervention, patients recorded their well-being with two different IBS-related and one health-related quality of life questionnaires, namely the IBS Symptom Severity Scale (IBS-SSS), Irritable Bowel Syndrome-Quality of Life (IBS-QOL), and the European Quality of Life-5 Dimensions (EQ-5D) questionnaires. The severity of gastrointestinal symptoms in all subjects was evaluated with the IBS-SSS. The IBS-SSS contains five questions that measure on a 100-point VAS the severity of abdominal pain, the frequency of abdominal pain, the severity of abdominal distension, the dissatisfaction with bowel habits, and the interference with quality of life [23]. All five components contribute to the score equally yielding a theoretical range of 0–500, with a higher score indicating worse condition. Scores below 75 represent controls and patients in remission, 75–175 represent mild IBS symptoms, 175–300 represent moderate severity, and scores above 300 represent severe IBS. The total severity score on the IBS-SSS is considered the gold standard measure of IBS severity. In the study by Francis et al., a decrease of 50 points correlated with improvement in clinical symptoms [23]. The IBS-QOL is a well-established 34-item measure assessing the degree to which IBS interferes with patient quality of life. Each item is rated on a five-point Likert scale, thus yielding a total score that has a theoretical range of 34 to 170 with higher scores indicating worse QOL, but which is then converted to a scale from 0 to 100 in which 0 reflects the worst and 100 the best possible condition [24, 25]. The IBS-SSS and the IBS-QOL were complemented by the EQ-5D questionnaire, a well-established standardized instrument for self-completion for the measure of health outcome in variable health conditions. It consists of three single questions that allow three different answers and a 100-point VAS on which 0 reflects the worst and 100 the best possible condition. All questionnaires have been used in German language before [26, 27]. In addition, stool frequency was quantified before and at the end of the study.

Before the dietary intervention, blood was collected for genotyping and serum was isolated and frozen at −20 °C until analysis. The expression of HLA-DQ2 (DQA1*0501/B1*0201 or DQA1*0505/DQB1*0202) and of HLA-DQ8 (DQA1*03/B1*0302) was determined using a reverse hybridization kit (RDB2105, AID Diagnostika GmbH, Strassberg, Germany) according to the manufacturer’s instructions. The determination of HLA-DQ2/8 followed the dietary intervention to avoid bias, and no clinical information was available to the performers and readers of the test.

At the end of the dietary intervention, patients were left to feel free to continue the GFD and were contacted 1 year after initiation of the GFD to determine their long-term dietary habits after the intervention with the SGA of relief.

### Definition of responders

The SGA of relief was the main efficacy variable. Patients who answered “considerably relieved” or “completely relieved” on at least 75 % of weeks over the 4 months of dietary intervention were defined as responders. These response criteria took into account the magnitude of effect (considerably relieved or completely relieved) and persistence of effect (75 % of the time). We used the stricter requirement “75 % of time” instead of “50 % of time,” which is commonly used in pharmaceutical studies, because we assumed that symptoms should disappear under a GFD when WS is their primary cause. Nevertheless, the weaker response criterion 50% of time was used as a secondary outcome measure.

Other secondary outcome measures included changes in the score of the IBS-QOL, IBS-SSS, and EQ-5D questionnaires and changes in pain intensity, stool consistency, and stool frequency before and at the end of the study.

### Statistical analysis

Data are described as proportions or as median (10th percentile-90th percentile) for continuous variables. To evaluate the predictive ability of HLA-DQ2/8 expression to discriminate between patients with and without WS, we determined sensitivity and specificity including 95 % confidence intervals. According to the recommended method by Krummenauer and Kauczor [28], a total number of 99 patients is required to construct a 95 % confidence interval with length of 20 % for an expected specificity of 90 % and a prevalence of WS of approx. 35 %. The study was terminated early because of difficulty with recruitment of patients fulfilling the ROME III criteria and willing to follow a GFD. Additional outcome variables (e.g., IBS-QoL score, EQ-5D score, IBS-SSS) were compared between responders and non-responders using the non-parametric Mann-Whitney *U* test, and values of *p* < 0.05 were considered statistically significant.

## Results

### Patients characteristics

The subjects (1092) followed the study call for participation and contacted the study office between February 2012 and March 2015. Of these, 91 fulfilled the primary inclusion criteria (Rome III criteria, self-reported weekly symptoms) for IBS-D or -M and were eligible to enter the 4-week observation period. Forty-five subjects did not finish the observation period: 15 participants due to “significant improvement” or “symptoms less than once a week,” 30 participants due to other reasons (classified as dropouts in Fig. 1). The most common reasons were “personal reasons” (*n* = 12) followed by “concerns about expenses, complexity and taste of a GFD” (*n* = 10), “external determination of HLA-DQ2/8 status during observation period” (*n* = 4), “long distance from study center” (*n* = 3), “being on a GFD” (*n* = 1). Eleven patients dropped out due to a new diagnosis: celiac disease (*n* = 3), lactose intolerance (*n* = 3), pancreatic insufficiency (*n* = 1), erosive duodenitis (*n* = 1), pregnancy (*n* = 1), giardiasis (*n* = 1), and mastocytosis (*n* = 1).

Thirty-five patients entered the study whose baseline characteristics are presented in Table 1. The proportion of IBS-D and IBS-M was balanced within the study cohort. Three out of four patients were female with no relevant gender differences among the subgroups and all patients were of Caucasian origin. The mean age was not different among the two subgroups. Forty percent of the study population was positive for either the HLA-DQ2 or the -DQ8 allele with a proportion of 2.5:1. This represents slightly more than in the general population [29]. There was a significantly higher proportion of HLA-DQ2/8 positive patients in the IBS-M than in the IBS-D subgroup (10 (50 %) vs. 4 (27 %), *p* < 0.05).

**Table 1:** Baseline characteristics

	IBS-D	IBS-M	Total
No. of patients	15 (43 %)	20 (57 %)	35
Age, mean (range)	47.1 (28–62)	50.2 (27–66)	48.9 (27–66)
Gender			
Male	5 (33 %)	4 (20 %)	9 (26 %)
Female	10 (67 %)	16 (80 %)	26 (74 %)
Symptom severity (mean ± SD)			
IBS-QOL	47.4 ± 16.0	53.9 ± 21.6	50.7 ± 18.9
IBS-SSS	290.5 ± 72.8	276.8 ± 79.7	290.7 ± 74.8
EQ-5D VAS (mm)	45.6 ± 24.5	53.5 ± 30.0	49.7 ± 27.2
Stools per day (mean ± SD)	4.2 ± 3.5	2.3 ± 1.6	3.5 ± 2.8
HLA-DQ2 positive	3 (20 %)	7 (35 %)	10 (29 %)
HLA-DQ8 positive	1 (7 %)	3 (15 %)	4 (11 %)
HLA-DQ2/-DQ8 negative	11 (73 %)	10 (50 %)	21 (60 %)

As assessed by the dietician, 33 of 35 patients (94 %) adhered to the GFD for the time of the study. Adverse events were not observed, but one female HLA-DQ2-positive IBS-M patient stopped the GFD after 5 weeks because of increased symptom severity and one male HLA-DQ2/8-negative IBS-M patient dropped out after 6 weeks due to a new steroid treatment for other reasons. Both had not reported improvement of symptoms and were therefore classified as non-responders.

### Wheat sensitivity and response to GFD

Twelve of the 35 patients (34 %; 95 % CI 21–51 %) that completed the study reported considerably relieved or completely relieved on at least 75 % of weeks over the 4 months of dietary intervention and were therefore classified as responders having WS. Of these 12 patients, three were completely relieved after 4 months. The proportion of responders was similar in the two IBS subgroups (Table 2).

**Table 2:** Responder by HLA-DQ2/8 status and by IBS subtype

	HLA-DQ2/8 positive	HLA-DQ2/8 negative	IBS-D	IBS-M
Responder	3 (21 %)	9 (43 %)	5 (33 %)	7 (35 %)
Non-responder	11 (79 %)	12 (57 %)	10 (67 %)	13 (65 %)

### Association of HLA-DQ2/8 status with wheat sensitivity

HLA-DQ2 or HLA-DQ8 expression was not associated with WS. In fact, the proportion of responders to GFD was even higher in patients negative for HLA-DQ2 or HLA-DQ8 (Table 2). Sensitivity and specificity of HLA-DQ2 or -DQ8 expression for WS were only 25 % (8–54 %) and 52 % (33–71 %), respectively. Thus, HLA-DQ2 or HLA-DQ8 expression did not discriminate IBS patients with and without WS in our cohort.

### Quality of life measures in responders and non-responders to GFD

**Fig. 2:**
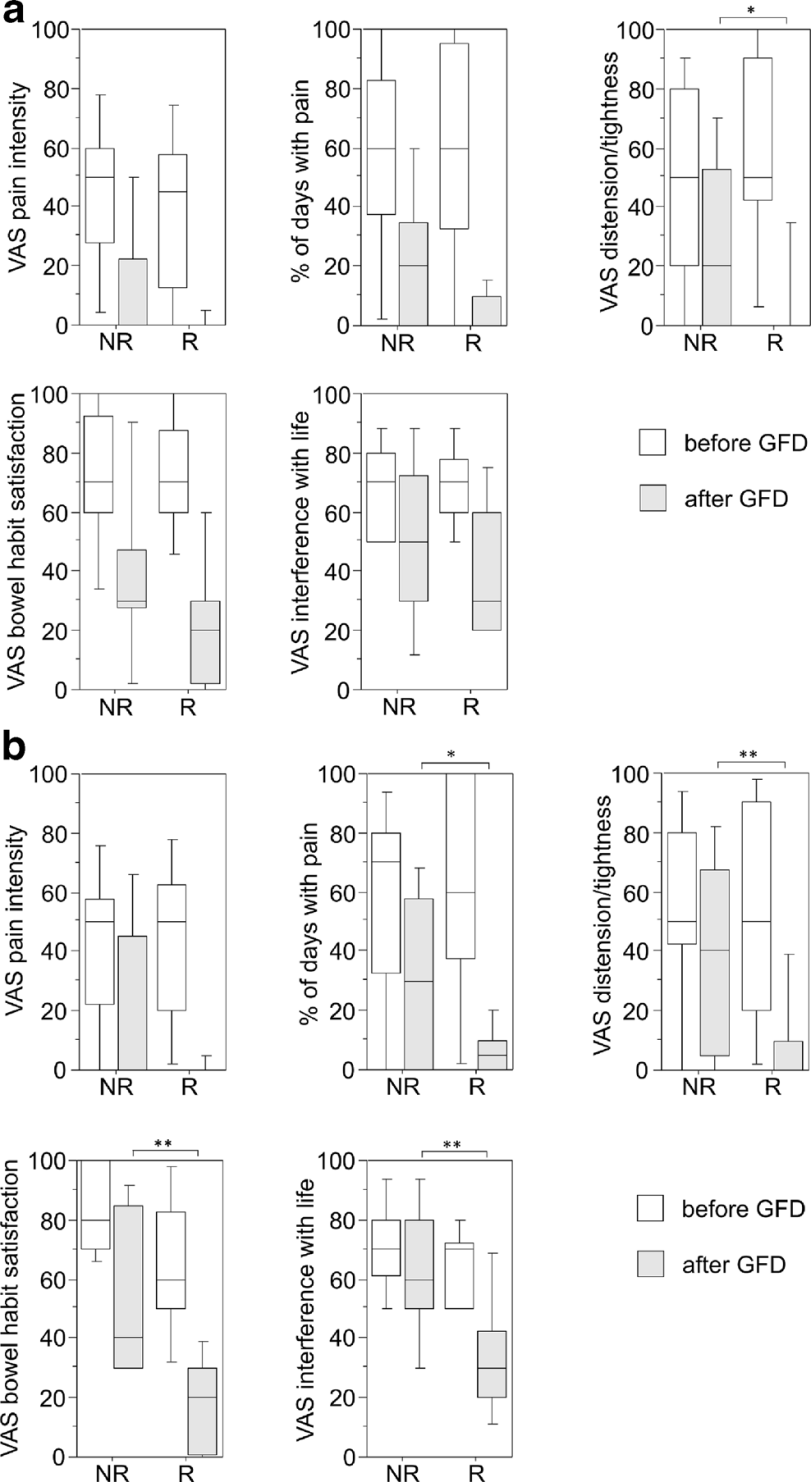
Results of the secondary outcome measures obtained by the IBS-SSS. **a** Comparison of responder (*R*) versus non-responder (*NR*) of the 75 % criterion for the indicated measures. Data are presented as *box plots* for *n* = 12 R and n = 19 NR. *p < 0.05. **b** Comparison of responder (*R*) versus non-responder (*NR*) of the 50 % criterion for the indicated measures. Data are presented as *box plots* for *n* = 18 R and *n* = 13 NR. *p < 0.05, **p < 0.005

In order to ascertain the validity of the SGA of relief as response measurement, we evaluated the three questionnaires IBS-SSS, IBS-QOL, and EQ-5D before and after the intervention and compared responders and non-responders using both our strict criterion (considerably relieved or completely relieved on at least 75 % of weeks) and the standard criterion (considerably relieved or completely relieved on at least 50 % of weeks). The results are demonstrated in Table 3 and Fig. 2a, b.

**Table 3:** Comparison of the IBS-SSS, IBS-QOL and the EQ-5D VAS score in the R75/NR75 group (A) and the R50/NR50 group (B)

A						
	IBS-SSS		IBS-QOL		EQ-5D VAS	
	Before GFD	After GFD	Before GFD	After GFD	Before GFD	After GFD
R75	275 (212–384) (*n* = 11)	70 (29–146) (*n* = 9)	53 (25–69) (*n* = 11)	78 (55–97) (*n* = 9)	40 (4–83) (*n* = 12)	90 (64–99) (*n* = 11)
NR75	280 (212–402) (*n* = 17)	130 (42–362) (*n* = 17)	51 (21–80) (*n* = 17)	72 (31–91) (*n* = 17)	60 (16–75) (*n* = 19)	80 (31–95) (*n* = 19)
P	n.s.	<0.05	n.s.	n.s.	n.s.	n.s.
B						
R50	275 (202–400) (*n* = 17)	50 (22–150) (*n* = 15)	59 (23–81) (*n* = 17)	81 (60–97) (*n* = 16)	63 (10–80) (*n* = 18)	90 (58–98) (*n* = 17)
NR50	290 (232–414) (*n* = 11)	175 (92–394) (*n* = 11)	50 (19–67) (*n* = 11)	54 (26–80) (*n* = 11)	33 (8–90) (*n* = 13)	75 (26–92) (*n* = 13)
P	n.s.	<0.001	n.s.	<0.01	n.s.	<0.05

Data are given as median with range (10th and 90th percentile)

In the responder group (R) fulfilling the 75 % criterion (R75), five out of nine patients reported an overall score of the IBS-SSS below 75 after 4 months of GFD (Table 3A). This score reflects that these patients turned into remission while being on the diet. There was a marked difference compared to the group of non-responders with only 4 out of 17 patients reporting a score below 75 after 4 months of GFD (Table 3A). The others still presented with mild to moderate and severe symptoms. Similarly, the overall score of the IBS-QOL and the results from the VAS of the EQ-5D revealed a tendency toward improvement. However, statistical significance was not reached in either of the two tests (Table 3A). When comparing the two relevant IBS parameters pain and distension/tightness obtained from the IBS-SSS before and after the intervention, only distension/tightness showed a significant improvement in the R75 group versus the NR75 group, whereas pain, defined as pain intensity on a VAS and occurrence of pain defined as % of days with abdominal pain, exhibited only a tendency toward improvement not reaching statistical significance (Fig. 2a). The same observation was made for “satisfaction with bowel habits” and “interference with life in general” (Fig. 2a).

When applying the less strict requirement of reporting considerably relieved or completely relieved on at least 50 % of weeks as recommended for pharmaceutical studies 18 of 34 patients (51 %) were responders. Also, in this group, the correlation with the secondary outcome measures was consistent but the differences were more pronounced (Table 3B). In the responder group (R50), an IBS-SSS overall score below 75 was reported by 9 out of 15 patients after 4 months of GFD. In parallel, the IBS-QOL overall score and the EQ-5D VAS score increased (Table 3B). In all three questionnaires, a significant symptom improvement could be detected after the diet in comparison to the group of non-responders (NR50; Table 3B). Corresponding results were obtained for the occurrence of pain, abdominal distension/tightness, satisfaction with bowel habits, and interference with life in general (Fig. 2b).

Similar observations were made for the IBS-QOL subscales. More pronounced and statistically significant differences were seen in the 50 % of time criterion than in the 75 % of time criterion. The detailed results of the IBS-QOL subscales are demonstrated in the supplementary material.

Stool frequency changed neither in IBS-D nor in IBS-M responder and revealed only a tendency to decrease in IBS-D. Stool frequency in the R75 group lowered from 3.8 ± 1.9 (*n* = 5) to 1.9 ± 1.3 (*n* = 4; *p* = n.s.) stools per day in IBS-D patients, whereas it was 2.5 ± 2.1 (*n* = 6) before and 1.7 ± 1.2 (*n* = 6; *p* = n.s.) stools per day after the diet in IBS-M patients. In R50 IBS-D patients stool frequency changed from 3.3 ± 1.9 (*n* = 7) stools per day to 1.7 ± 1.1 (*n* = 6; *p* = n.s.) and was 2.5 ± 1.7 (*n* = 10) before and 1.7 ± 1.2 (*n* = 10; *p* = n.s.) stools per day after the diet in IBS-M patients. When comparing the two different response criteria and the corresponding secondary outcomes, in both groups, a significant symptom and quality of life improvement were observed after 4 months of GFD, but the results obtained from the 50 % of time criterion were more pronounced than that obtained from the 75 % of time criterion.

### GFD duration until clinical response

Interestingly, a considerable number of patients (six IBS-D (one DQ2+), seven IBS-M (three DQ2+/one DQ8+) presented with a delayed response to the GFD. These patients started to be considerably relieved or completely relieved only after approximately 2 months but reached a stable plateau hereafter. These patients formally were classified as non-responders when applying the 75 % of time criterion, and seven patients (four IBS-D (zero DQ2/8+) and three IBS-M (zero DQ2/8+)) were even non-responders when applying the 50 % of time criterion because of the late onset of relief.

**Fig. 3:**
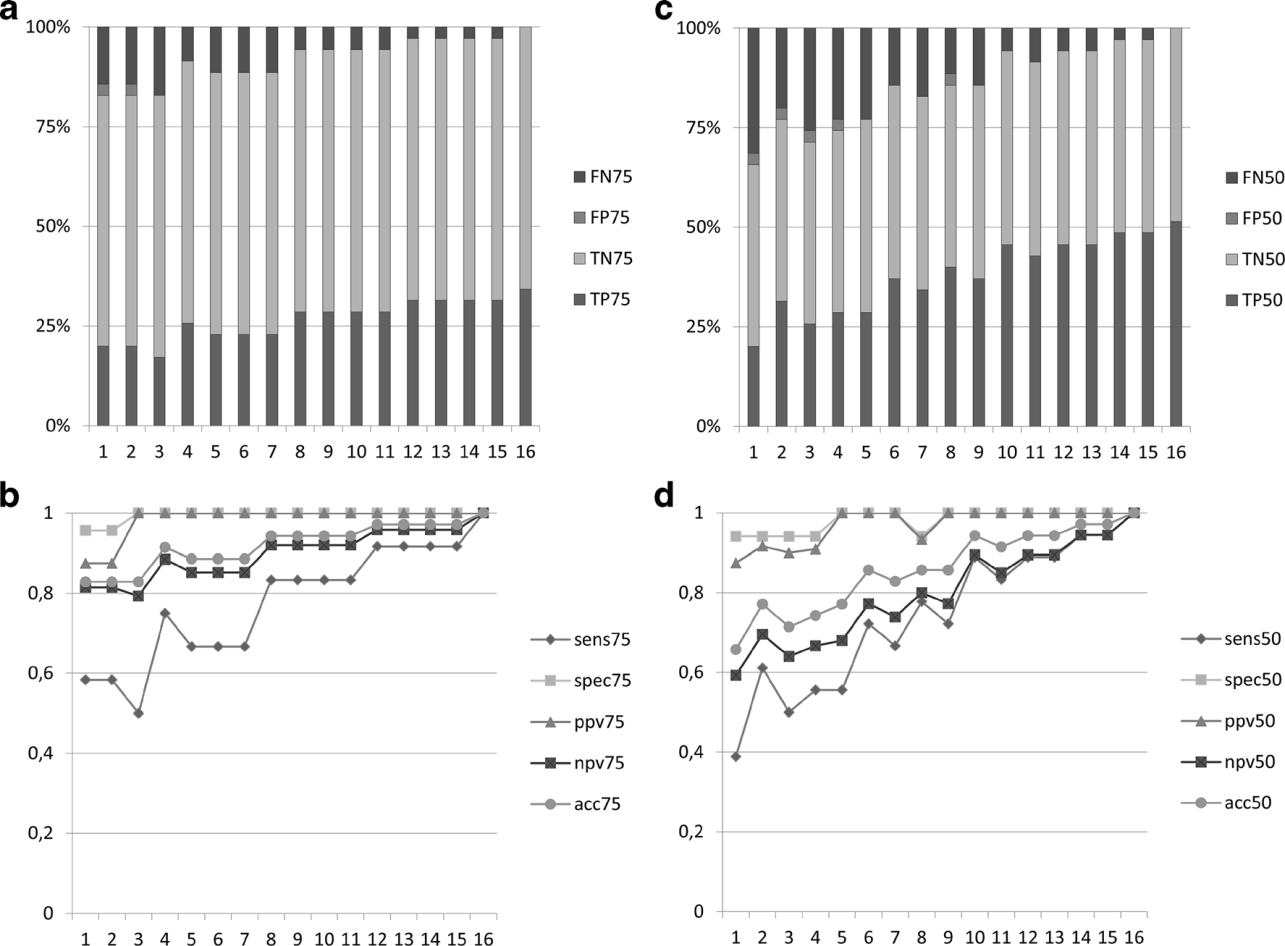
Core statistical parameters of the study. Diagram of the false negative (*FN*), false positive (*FP*), true negative (*TN*), and true positive (*TP*) values in relation to the primary outcome after 4 months of GFD (TN and TP set as 100 %) for the 75 % criterion (**a**) and the 50 % criterion (**c**). Diagram of sensitivity (*sens*), specificity (*spec*), positive predictive value (*ppv*), negative predictive value (*npv*), and accuracy *(acc*) for the 75 % criterion (**b**) and the 50 % criterion (**d**)

This observation is demonstrated statistically in Fig. 3a, b. When acting on the assumption that the primary outcome after 4 months of GFD is either true negative (TN) or true positive (TP), specificity in the 75 % of time criterion group after 2 months of GFD (spec75) is 100 % and sensitivity (sens75) is 83 % with a negative predictive value (npv75) of 92 %, a positive predictive value (ppv75) of 100 % and an accuracy (acc75) of 94 %.

Similar results were obtained in the 50 % of time criterion group after 2 months of GFD (Fig. 3c, d). Sensitivity (sens50) and specificity (spec50) were 78 and 94 % with a negative (npv50) and positive predictive value (ppv50) of 80 and 93 % and an accuracy (acc50) of 86 %. In this group, the placebo effect is also almost negligible with only one false positive (FP) individual after 2 months.

### One year follow-up

Twelve months after the start of the GFD, all patients were contacted to evaluate their long-term dietary habits in combination with the SGA of relief. The results are depicted in Table 4. In the responder group (R75), 7 out of 11 patients (64 %) still adhered to a strict GFD. Of these patients, two reported to be still completely relieved, four patients still felt considerably relieved, and one patient reported unchanged. The other four patients (36 %) from the responder group reported to feel considerably relieved. Of note, all of them continued to follow a GFD with some exceptions.

**Table 4:** Follow-up of dietary habits after 1 year in the R75 and NR75 group

	Strict GFD	Exceptions from strict GFD	No GFD	total
Responder	7 (64 %)	4 (36 %)	0 (0 %)	11
Non-responder	6 (33 %)	4 (22 %)	8 (44 %)	18

In the non-responder group, surprisingly, six patients (33 %) still followed a strict GFD. Three patients reported to feel considerably relieved, one was still “somewhat relieved” and two patients felt unchanged. Two of the three patients that reported to feel considerably relieved were part of the R50 group; the other patient that felt considerably relieved exhibited a good response to the GFD only in the last 2 weeks of the dietary intervention and was therefore originally classified as non-responder. Four patients (22 %) followed a GFD with some exceptions (considerably relieved 1; somewhat relieved 1; unchanged 2) and eight patients (44 %) did not follow the GFD (considerably relieved 2; somewhat relieved 3; unchanged 3). Of four patients, no sufficient information was gathered (1 responder, 3 non-responders).

These data illustrate a consistent adherence to the GFD in the responder group with long-term improvement of clinical symptoms.

## Discussion

In the last few years, efforts have been made to define non-celiac gluten sensitivity at three consensus conferences [30–32]. It was concluded that non-celiac gluten sensitivity is “a clinical entity induced by the ingestion of gluten leading to intestinal and/or extra-intestinal symptoms that resolve once the gluten-containing foodstuff is eliminated from the diet, and when celiac disease and wheat allergy have been ruled out” [17]. Since it is not clear whether gluten itself or other food or wheat components account for these symptoms as demonstrated lately for fermentable oligo-di-monosaccharides and polyols (FODMAPs) and amylase trypsin inhibitors (ATI) [16, 18], the term WS was preferred recently. In any case, the definition above suggests that a certain number of patients suffering from IBS might instead have WS and that removal of wheat from the diet would lead to a significant symptom improvement in these patients. However, the distribution of WS within patients fulfilling the Rome III criteria for IBS and in whom celiac disease and wheat allergy have been excluded is not clear.

In our study, 34 % of all IBS-D and IBS-M patients were responders to GFD according to our strict 75 % response criterion, independent of the IBS subtype, and were therefore classified as having WS. This proportion increased to 51 % when applying the less strict requirement of being considerably relieved or completely relieved on at least 50 % of weeks, which is common for pharmaceutical studies. In parallel, secondary outcome measures about disease- and health-related quality of life and IBS-related symptom scores improved. Using a 50-point improvement of the IBS-SSS as criterion, even 88 % of our IBS patients were responders in accordance with recent findings in IBS-D patients [33]. We expected that many IBS patients would show a placebo response which would disappear over time. Surprisingly, this was not the case and early responses were usually stable. On the other hand, several late responders were seen, and the proportion of false negative non-responders fell below 10 % not before 2 months. Our data thus support the current recommendation of 8–12 weeks observation in IBS therapy studies. Expression of HLA-DQ2 or -DQ8 did not discriminate patients with and without WS in our study. In fact, WS was even more common in the absence of these markers. An association between HLA-DQ2 expression and the response to GFD has been reported in some [15, 34, 35] but not all studies of IBS patients [33, 36]. Although the patient numbers studied so far are rather small, these observations indicate that WS may rather not be as closely related to celiac disease as previously suggested [14, 35]. However, potential celiac disease might be present in a subgroup of IBS patients characterized by predominant diarrhea, increased celiac-associated antibodies apart from anti-TTG IgA in serum and in duodenal succus, increased mucosal intraepithelial lymphocytes, HLA-DQ2 expression, and response to a GFD [14].

Thus, three important implications result from these observations: (i) WS is common within the group of IBS-D and IBS-M patients; (ii) WS is not associated with the HLA-DQ2/8 status; and (iii) long-term GFD seems to be necessary to identify all responders.

Although it is generally claimed that removal of gluten from the diet leads to symptom improvement in some IBS patients, studies systematically examining the effect of GFD on IBS symptoms are rare. To the best of our knowledge, there are only three studies that evaluated directly the effect of a GFD in IBS patients and only in the IBS-D subtype [14, 15, 33]. In two of these studies, it was suggested that HLA-DQ2 in conjunction with serum gliadin IgG antibodies might predict a response of stool frequency to a GFD in IBS-D patients [14, 15]. A recent study, however, revealed improvement of the IBS-SSS in 71 % of IBS-D patients independent of their HLA-DQ2/8 status [33]. Others recently applied crossover designs with gluten-free or gluten-containing food to examine the effects on IBS symptoms [16, 35, 37]. However, the duration of the elimination diet in these studies did not exceed 4–6 weeks, which is important when considering that gluten removal from the diet for such a short interval may not identify all responders. Late responders are missed and accordingly, the effect of a GFD may be underestimated in comparison to long-term studies [14, 15].

Interestingly, we observed two response characteristics within the responder group, namely (i) early responders that profited immediately after starting the GFD and (ii) late responders only benefitting after 2 months. On the one hand, this finding explains why the symptom improvements as determined by the secondary outcome measures between the R75 and the NR75 groups that contain the R50 patients are less pronounced than between the R50 and NR50 groups. On the other hand, this observation indicates that two different mechanisms may underlie gastrointestinal symptom improvement. Conflicting data exist about what food ingredients cause intestinal symptoms in WS. Carroccio et al. demonstrated in a double-blind placebo-controlled trial that addition of wheat to an elimination diet increased gastrointestinal symptoms in IBS patients and suggested the existence of non-celiac wheat sensitivity [38]. Biesiekierski et al. reported in 2011 that IBS patients that were symptomatically controlled on a GFD exhibited worsening of symptoms when placed on a gluten-containing diet for 6 weeks in a double-blind placebo controlled fashion and concluded that non-celiac gluten intolerance may exist [39]. In a following study 2 years later, the same group found evidence for this effect not to be specific for gluten. In patients with non-celiac gluten sensitivity that were placed on diets low in FODMAPs, the addition of gluten did not cause worsening of gastrointestinal symptoms. They concluded that rather FODMAPs than gluten are responsible for IBS-related symptoms in patients classified as having non-celiac gluten sensitivity and questioned the existence of non-celiac gluten sensitivity [16]. In our study, we cannot exclude that FODMAPs, which are naturally restricted in a GFD, are the cause for the symptom improvement in our responder group. We also did not perform a controlled re-challenge. However, the early effect we observed in the responder group may be attributed to FODMAPs, since its time course is consistent with the observations by Biesiekierski et al. [16]. The late response might be related to immunologic phenomena, possibly through antigenic components as, e.g., ATI, which have been demonstrated to cause an intestinal inflammatory reaction [18]. In any case, this cannot be solved because of missing biomarkers.

Independent of these findings, the results indicate that more research is needed in adverse reactions to food. In a cross-sectional study in Berlin, Germany, 34.9 % of patients reported to suffer from various kinds of adverse reactions to food. Double-blind-placebo-controlled testing and classical allergy testing could account for 3.6 % [40]. However, in other patients, it was difficult to say if there was truly no correlation. As emphasized by the present study, a diet of eight or more weeks might be necessary in many patients to see a positive response.

This underlines the necessity for novel diagnostic procedures to investigate the role of non-allergic hypersensitivity reactions to various food ingredients. Hence, at this point, we cannot exclude that IBS patients not reacting to wheat might still present with intolerant reactions to other food ingredients.

A recent study points toward a novel diagnostic measure. Here, the duodenal mucosa was challenged with potential antigens, and the subsequent cellular reaction was monitored via confocal endomicroscopy thus allowing for in vivo identification of food reactions [41].

In conclusion, our data provide evidence that a GFD offers relief for a particular group of non-constipated IBS patients. However, we could not show an association of response to a GFD and HLA-DQ2/DQ8 status and therefore cannot recommend determining HLA-DQ2/8 as a predictive marker for WS. Regardless, we believe that based on our data, it is justifiable to recommend a GFD in non-constipated IBS patients as a therapeutic option.

## Electronic supplementary material

ESM 1 (PDF 12 kb)
