# Supplementary material for: Long-term response to gluten-free diet as evidence for non-celiac wheat sensitivity in one third of patients with diarrhea-dominant and mixed-type irritable bowel syndrome
Source: Int J Colorectal Dis. 2016 Sep 30;32(1):29–39. doi: 10.1007/s00384-016-2663-x (PMC5219884; doi:10.1007/s00384-016-2663-x)
Supplement: Supplementary file 1 — (PDF 12 kb) [file 384_2016_2663_MOESM1_ESM.pdf]

SUPPLEMENTARY MATERIAL

Results of the IBS-QOL subscales

Supplementary Table 1: Responder 75 before and after four months of GFD

IBS-QoL subscale	Before GFD (n=11)	After GFD (n=9)	P
**Dysphoria**	**44.4±20.5**	**81.6±23.6**	**0.008**
**Interference with activity**	**48.4±11.6**	**77.1±16.3**	**0.008**
**Body image**	**44.9±23.0**	**81.9±19.1**	**0.008**
**Health worry**	**65.9±16.4**	**88.3±11.9**	**0.012**
Food avoidance	33.3±28.6	44.2±24.3	n.s.
**Social reaction**	**56.3±27.8**	**76.9±24.6**	**0.028**
sexual	70.5±32.7	86.1±18.6	n.s.
relationships	67.4±27.0	81.3±17.2	n.s.
**total**	**51.1±16.3**	**77.6±16.4**	**0.011**

Supplementary Table 2: Non-responder 75 before and after four months of GFD

IBS-QoL subscale	Before GFD (n=17)	After GFD (n=17)	P
**Dysphoria**	**39.5±21.8**	**59.0±29.5**	**0.003**
**Interference with activity**	**51.5±28.5**	**64.5±30.3**	**0.020**
**Body image**	**52.9±21.7**	**71.1±25.9**	**0.012**
Health worry	59.1±25.6	67.9±19.2	n.s.
Food avoidance	40.0±33.2	44.6±24.6	n.s.
**Social reaction**	**50.6±24.0**	**64.6±28.3**	**0.005**
sexual	75.0±25.8	79.4±21.6	n.s.
relationships	57.8±30.4	72.1±25.7	n.s.
**total**	**50.4±21.0**	**64.2±23.1**	**0.025**

Supplementary Table 3: Responder (R75) versus Non-responder 75 (NR75) after four months of GFD

IBS-QoL subscale	R75 (n=9)	NR75 (n=17)	P
**Dysphoria**	**81.6±23.6**	**59.0±29.5**	**0.049**
Interference with activity	77.1±16.3	64.5±30.3	n.s.
Body image	81.9±19.1	71.1±25.9	n.s.
**Health worry**	**88.3±11.9**	**67.9±19.2**	**0.008**
Food avoidance	44.2±24.3	44.6±24.6	n.s.
Social reaction	76.9±24.6	64.6±28.3	n.s.
sexual	86.1±18.6	79.4±21.6	n.s.
relationships	81.3±17.2	72.1±25.7	n.s.
total	77.6±16.4	64.2±23.1	n.s.

Supplementary Table 4: Responder 50 before and after four months of GFD

IBS-QoL subscale	Before GFD (n=17)	After GFD (n=16)	P
**Dysphoria**	**47.3±20.0**	**83.6±19.4**	**0.001**
**Interference with activity**	**54.4±22.2**	**81.7±15.0**	**0.001**
**Body image**	**48.2±20.6**	**85.2±15.3**	**0.001**
**Health worry**	**66.2±21.5**	**82.3±17.4**	**0.024**
Food avoidance	40.2±34.6	47.4±20.3	n.s.
**Social reaction**	**58.1±25.1**	**79.7±20.6**	**0.004**
sexual	74.3±31.7	89.2±18.2	n.s.
relationships	67.2±27.6	82.6±16.5	n.s.
**total**	**54.5±19.0**	**79.8±13.9**	**0.001**

Supplementary Table 5: Non-responder 50 before and after four months of GFD

IBS-QoL subscale	Before GFD (n=11)	After GFD (n=11)	P
Dysphoria	32.3±20.2	43.8±23.9	n.s.
Interference with activity	43.8±24.0	51.0±28.9	n.s.
Body image	52.3±25.2	60.4±26.3	n.s.
Health worry	54.9±22.9	65.5±18.1	n.s.
Food avoidance	33.0±25.6	40.2±28.1	n.s.
Social reaction	44.7±24.4	53.8±28.1	n.s.
sexual	71.6±23.1	71.6±22.4	n.s.
relationships	53.0±30.4	65.2±27.8	n.s.
total	44.8±18.1	53.7±21.1	n.s.

Supplementary Table 6: Responder (R50) versus Non-responder 50 (NR50) after four months of GFD

IBS-QoL subscale	R50 (n=16)	NR50 (n=11)	P
**Dysphoria**	**83.6±19.4**	**43.8±23.9**	**0.000**
**Interference with activity**	**81.7±15.0**	**51.0±28.9**	**0.008**
**Body image**	**85.2±15.3**	**60.4±26.3**	**0.010**
**Health worry**	**82.3±17.4**	**65.5±18.1**	**0.030**
Food avoidance	47.4±20.3	40.2±28.1	n.s.
**Social reaction**	**79.7±20.6**	**53.8±28.1**	**0.013**
**sexual**	**89.2±18.2**	**71.6±22.4**	**0.026**
relationships	82.6±16.5	65.2±27.8	n.s.
**total**	**79.8±13.9**	**53.7±21.1**	**0.003**

All data are given as mean±SD. P< 0.05 was considered significant.
